# Correction: Influence of palliative care policy on place of death for people with different cancer types: a nationwide’ register study

**DOI:** 10.1371/journal.pone.0339991

**Published:** 2025-12-31

**Authors:** Joakim Öhlén, Stina Nyblom, Anneli Ozanne, Stefan Nilsson, Hanna Gyllensten, Anna O’Sullivan, Carl Johan Fürst, Cecilia Larsdotter

The first and last names of authors two to eight are incorrectly switched. The correct names are: Stina Nyblom, Anneli Ozanne, Stefan Nilsson, Hanna Gyllensten, Anna O’Sullivan, Carl Johan Fürst, Cecilia Larsdotter. The correct citation is: Öhlén J, Nyblom S, Ozanne A, Nilsson S, Gyllensten H, O’Sullivan A, et al. (2025) Influence of palliative care policy on place of death for people with different cancer types: a nationwide’ register study. PLoS ONE 20(3): e0320086. https://doi.org/10.1371/journal.pone.0320086.
