# Correction: Influence of palliative care policy on place of death for people with different cancer types: A nationwide’ register study

**DOI:** 10.1371/journal.pone.0336828

**Published:** 2026-01-07

**Authors:** Joakim Öhlén, Stina Nyblom, Anneli Ozanne, Stefan Nilsson, Hanna Gyllensten, Anna O’Sullivan, Carl Johan Fürst, Cecilia Larsdotter

The author initials appear incorrectly in the citation. The correct citation is: Öhlén J, Nyblom S, Ozanne A, Nilsson S, Gyllensten H, O’Sullivan A, et al. (2025) Influence of palliative care policy on place of death for people with different cancer types: a nationwide’ register study. PLoS ONE 20(3): e0320086. https://doi.org/10.1371/journal.pone.0320086.

The first and last name of the six, seventh, and eighth authors are incorrectly switched. The correct names are: Anna O’Sullivan, Carl Johan Fürst, Cecilia Larsdotter.
