# Supplementary material for: Influence of palliative care policy on place of death for people with different cancer types: a nationwide’ register study
Source: PLoS One. 2025 Mar 27;20(3):e0320086. doi: 10.1371/journal.pone.0320086 (PMC11949374; doi:10.1371/journal.pone.0320086)
Supplement: S1 Table — (PDF) [file pone.0320086.s001.pdf]

Supplementary Table 1. Cross-regional population characteristics

Variable	Healthcare Regions						
	Total (n=152414)	Uppsala-Örebro region (n=34793)	Northern region (n=15142)	Stockholm region (n=28487)	Western region (n=27611)	Southeastern region (n=17481)	Southern region (n=28900)
**Sex**							
**Male**	79,502 (52.2%)	18,459 (53.1%)	7,902 (52.2%)	14,367 (50.4%)	14,432 (52.3%)	9,191 (52.6%)	15,151 (52.4%)
**Female**	72,912 (47.8%)	16,334 (46.9%)	7,240 (47.8%)	14,120 (49.6%)	13,179 (47.7%)	8,290 (47.4%)	13,749 (47.6%)
**Age at death, years**							
**60-69**	28,626 (18.8%)	6,462 (18.6%)	2,818 (18.6%)	5,572 (19.6%)	5,144 (18.6%)	3,127 (17.9%)	5,503 (19.0%)
**18-29**	424 (0.3%)	82 (0.2%)	32 (0.2%)	96 (0.3%)	77 (0.3%)	57 (0.3%)	80 (0.3%)
**30-39**	984 (0.6%)	190 (0.5%)	89 (0.6%)	250 (0.9%)	170 (0.6%)	106 (0.6%)	179 (0.6%)
**40-49**	3,310 (2.2%)	671 (1.9%)	273 (1.8%)	780 (2.7%)	633 (2.3%)	361 (2.1%)	592 (2.0%)
**50-59**	10,008 (6.6%)	2,099 (6.0%)	900 (5.9%)	2,127 (7.5%)	1,893 (6.9%)	1,132 (6.5%)	1,857 (6.4%)
**70-79**	49,644 (32.6%)	11,722 (33.7%)	5,004 (33.0%)	9,361 (32.9%)	8,717 (31.6%)	5,697 (32.6%)	9,143 (31.6%)
**80-89**	45,235 (29.7%)	10,395 (29.9%)	4,840 (32.0%)	7,639 (26.8%)	8,377 (30.3%)	5,252 (30.0%)	8,732 (30.2%)
**90+**	14,183 (9.3%)	3,172 (9.1%)	1,186 (7.8%)	2,662 (9.3%)	2,600 (9.4%)	1,749 (10.0%)	2,814 (9.7%)
**Cancer type**							
**Other**	18,962 (12.4%)	4,207 (12.1%)	1,958 (12.9%)	,3464 (12.2%)	3,508 (12.7%)	2,186 (12.5%)	3,639 (12.6%)
**Lower gastrointestinal**	18,696 (12.3%)	4,288 (12.3%)	1,877 (12.4%)	3,086 (10.8%)	3,526 (12.8%)	2,150 (12.3%)	3,769 (13.0%)
**Upper gastrointestinal**	29,684 (19.5%)	6,873 (19.8%)	3,219 (21.3%)	5,712 (20.1%)	5,176 (18.7%)	3,307 (18.9%)	5,397 (18.7%)
**Pulmonary**	24,755 (16.2%)	5,671 (16.3%)	2,116 (14.0%)	5,043 (17.7%)	4,263 (15.4%)	2,716 (15.5%)	4,946 (17.1%)
**Breast and gynaecological**	17,923 (11.8%)	3,879 (11.1%)	1,671 (11.0%)	3,671 (12.9%)	3,320 (12.0%)	2,087 (11.9%)	3,295 (11.4%)
**Prostate and urinary tract**	24,929 (16.4%)	6,002 (17.3%)	2,666 (17.6%)	4,243 (14.9%)	4,553 (16.5%)	2,938 (16.8%)	4,527 (15.7%)
**Haematological**	13,235 (8.7%)	2,932 (8.4%)	1,258 (8.3%)	2,497 (8.8%)	2,442 (8.8%)	1,593 (9.1%)	2,513 (8.7%)
**Malignant melanoma and sarcoma**	4,230 (2.8%)	941 (2.7%)	377 (2.5%)	771 (2.7%)	823 (3.0%)	504 (2.9%)	814 (2.8%)

Variable	Healthcare Regions						
	Total (n=152414)	Uppsala-Örebro region (n=34793)	Northern region (n=15142)	Stockholm region (n=28487)	Western region (n=27611)	Southeastern region (n=17481)	Southern region (n=28900)
**Living conditions, Number of children under 18**							
**No children under 18**	145,384 (95.7%)	33,415 (96.2%)	14,609 (96.6%)	26,662 (94.2%)	26,307 (95.5%)	16,751 (96.0%)	27,640 (96.0%)
**Children under 18**	6,572 (4.3%)	1,307 (3.8%)	517 (3.4%)	1,652 (5.8%)	1,239 (4.5%)	696 (4.0%)	1,161 (4.0%)
**Living in single-person household**							
**Single-person household**	61,584 (40.5%)	14,033 (40.4%)	6,101 (40.3%)	11,740 (41.5%)	10,897 (39.6%)	7,063 (40.5%)	11,750 (40.8%)
**Multi-person household**	90,372 (59.5%)	20,689 (59.6%)	9,025 (59.7%)	16,574 (58.5%)	16,649 (60.4%)	10,384 (59.5%)	17,051 (59.2%)
**Year of Death**							
**2013**	21,196 (13.9%)	4,895 (14.1%)	2,076 (13.7%)	3,880 (13.6%)	3,764 (13.6%)	2,494 (14.3%)	4,087 (14.1%)
**2014**	21,439 (14.1%)	4,855 (14.0%)	2,166 (14.3%)	3,853 (13.5%)	3,896 (14.1%)	2,519 (14.4%)	4,150 (14.4%)
**2015**	21,686 (14.2%)	4,974 (14.3%)	2,185 (14.4%)	4,140 (14.5%)	3,908 (14.2%)	2,468 (14.1%)	4,011 (13.9%)
**2016**	21,847 (14.3%)	5,025 (14.4%)	2,129 (14.1%)	4,097 (14.4%)	3,897 (14.1%)	2,447 (14.0%)	4,252 (14.7%)
**2017**	22,369 (14.7%)	5,094 (14.6%)	2,193 (14.5%)	4,133 (14.5%)	4,164 (15.1%)	2,583 (14.8%)	4,202 (14.5%)
**2018**	21,916 (14.4%)	4,911 (14.1%)	2,208 (14.6%)	4,256 (14.9%)	3,991 (14.5%)	2,489 (14.2%)	4,061 (14.1%)
**2019**	21,961 (14.4%)	5,039 (14.5%)	2,185 (14.4%)	4,128 (14.5%)	3,991 (14.5%)	2,481 (14.2%)	4,137 (14.3%)
**Marital status**							
**Married**	69,615 (45.7%)	15,754 (45.3%)	6,741 (44.5%)	12,472 (43.8%)	12,930 (46.8%)	8,256 (47.2%)	13,462 (46.6%)
**Unmarried**	19,480 (12.8%)	4,595 (13.2%)	2,207 (14.6%)	4,074 (14.3%)	3,328 (12.1%)	2,047 (11.7%)	3,229 (11.2%)
**Widowed**	36,492 (23.9%)	8,405 (24.2%)	3,796 (25.1%)	6,125 (21.5%)	6,569 (23.8%)	4,501 (25.7%)	7,096 (24.6%)
**Divorced**	26,827 (17.6%)	6,039 (17.4%)	2,398 (15.8%)	5,816 (20.4%)	4,784 (17.3%)	2,677 (15.3%)	5,113 (17.7%)
**Educational attainment**							
**Higher secondary education**	63,371 (42.2%)	14,378 (41.8%)	6,748 (44.8%)	12,561 (45.1%)	11,012 (40.5%)	6,847 (39.7%)	11,825 (41.6%)

Variable	Healthcare Regions						
	Total (n=152414)	Uppsala-Örebro region (n=34793)	Northern region (n=15142)	Stockholm region (n=28487)	Western region (n=27611)	Southeastern region (n=17481)	Southern region (n=28900)
**No formal or elementary education**	46,556 (31.0%)	11,755 (34.2%)	4,925 (32.7%)	5,432 (19.5%)	8,874 (32.7%)	6,329 (36.7%)	9,241 (32.5%)
**Lower secondary education**	13,857 (9.2%)	3,074 (8.9%)	1,123 (7.5%)	2,996 (10.8%)	2,627 (9.7%)	1,514 (8.8%)	2,523 (8.9%)
**Higher education**	26,309 (17.5%)	5,170 (15.0%)	2,259 (15.0%)	6,845 (24.6%)	4,647 (17.1%)	2,568 (14.9%)	4,820 (17.0%)
**Residing in urban area**							
**NO**	20,565 (13.5%)	5,919 (17.0%)	3,321 (21.9%)	1,533 (5.4%)	3,955 (14.3%)	2,593 (14.8%)	3,244 (11.2%)
**Residing in urban area**	131,849 (86.5%)	28,874 (83.0%)	11,821 (78.1%)	26,954 (94.6%)	23,656 (85.7%)	14,888 (85.2%)	25,656 (88.8%)
**Country of birth**							
**Born in Sweden**	132,549 (87.0%)	30,894 (88.8%)	14,174 (93.6%)	22,676 (79.6%)	23,956 (86.8%)	15,846 (90.7%)	25,003 (86.5%)
**Born outside Sweden**	19,860 (13.0%)	3,899 (11.2%)	968 (6.4%)	5,810 (20.4%)	3,654 (13.2%)	1,633 (9.3%)	3,896 (13.5%)
**Place of death within a specialised palliative care facility**							
**No**	97,071 (63.7%)	24,793 (71.3%)	10,647 (70.3%)	10,580 (37.1%)	20,071 (72.7%)	12,696 (72.6%)	18,284 (63.3%)
**Yes**	55,343 (36.3%)	10,000 (28.7%)	4,495 (29.7%)	17,907 (62.9%)	7,540 (27.3%)	4,785 (27.4%)	10,616 (36.7%)
**Palliative care diagnosis; ICD-code Z51.5**							
**No**	106,127 (69.6%)	26,896 (77.3%)	10,809 (71.4%)	12,992 (45.6%)	20,392 (73.9%)	11,352 (64.9%)	23,686 (82.0%)
**Yes**	46,287 (30.4%)	7,897 (22.7%)	4,333 (28.6%)	15,495 (54.4%)	7,219 (26.1%)	6,129 (35.1%)	5,214 (18.0%)

Notes. For categorical variables n (%) is presented. For continuous variables Mean (SD) /Median (Min; Max) / n= is presented. Regions with >1% difference than the total population are marked in yellow, and regions with >5% difference are marked in read.
