# Supplementary material for: Influence of palliative care policy on place of death for people with different cancer types: a nationwide’ register study
Source: PLoS One. 2025 Mar 27;20(3):e0320086. doi: 10.1371/journal.pone.0320086 (PMC11949374; doi:10.1371/journal.pone.0320086)
Supplement: S2 Table — (PDF) [file pone.0320086.s002.pdf]

Supplementary Table 2. **Utilisation of specialised palliative care services at the end-of-life from 2013 to 2019 by healthcare region and cancer types**

		Home death		Hospital death		Nursing home death		Death in other place [a]
		utilising specialised palliative care services		utilising specialised palliative care services		utilising specialised palliative care services		
**Cancer type**	**Healthcare region**	**No** [a, b]	**Yes** [a, c]	**No** [a, d]	**Yes** [a, e]	**No** [a, f]	**Yes** [a, g]	
Lower gastrointestinal	Northern region	235 (12.5%)	259 (13.8%)	580 (30.9%)	149 (7.9%)	427 (22.7%)	116 (6.2%)	111 (5.9%)
	Uppsala-Örebro region	664 (15.5%)	630 (14.7%)	1,162 (27.1%)	679 (15.8%)	1,094 (25.5%)	47 (1.1%)	12 (0.3%)
	Stockholm region	70 (2.3%)	550 (17.8%)	592 (19.2%)	1,286 (41.7%)	396 (12.8%)	41 (1.3%)	151 (4.9%)
	Western region	610 (17.3%)	250 (7.1%)	891 (25.3%)	449 (12.7%)	876 (24.8%)	401 (11.4%)	49 (1.4%)
	Southeastern region	423 (19.7%)	378 (17.6%)	455 (21.2%)	219 (10.2%)	583 (27.1%)	90 (4.2%)	2 (0.1%)
	Southern region	564 (15.0%)	642 (17.0%)	874 (23.2%)	570 (15.1%)	833 (22.1%)	155 (4.1%)	131 (3.5%)
	Total	2,567 (13.7%)	2,710 (14.5%)	4,560 (24.4%)	3,355 (17.9%)	4,209 (22.5%)	852 (4.6%)	456 (2.4%)
Upper gastrointestinal	Northern region	381 (11.8%)	444 (13.8%)	1,156 (35.9%)	306 (9.5%)	522 (16.2%)	228 (7.1%)	182 (5.7%)
	Uppsala-Örebro region	924 (13.4%)	1,019 (14.8%)	2,117 (30.8%)	1,386 (20.2%)	1,307 (19.0%)	94 (1.4%)	26 (0.4%)
	Stockholm region	145 (2.5%)	1,075 (18.8%)	1,147 (20.1%)	2,595 (45.4%)	388 (6.8%)	63 (1.1%)	299 (5.2%)
	Western region	934 (18.0%)	404 (7.8%)	1,518 (29.3%)	686 (13.3%)	974 (18.8%)	598 (11.6%)	62 (1.2%)
	Southeastern region	639 (19.3%)	588 (17.8%)	852 (25.8%)	347 (10.5%)	723 (21.9%)	153 (4.6%)	5 (0.2%)
	Southern region	594 (11.0%)	1,009 (18.7%)	1,373 (25.4%)	1,086 (20.1%)	932 (17.3%)	225 (4.2%)	178 (3.3%)
	Total	3,617 (12.2%)	4,539 (15.3%)	8,165 (27.5%)	6,407 (21.6%)	4,847 (16.3%)	1,361 (4.6%)	753 (2.5%)
Pulmonary	Northern region	221 (10.4%)	150 (7.1%)	938 (44.3%)	222 (10.5%)	356 (16.8%)	115 (5.4%)	114 (5.4%)
	Uppsala-Örebro region	695 (12.3%)	500 (8.8%)	2,293 (40.4%)	878 (15.5%)	1,210 (21.3%)	68 (1.2%)	27 (0.5%)
	Stockholm region	133 (2.6%)	658 (13.0%)	1,224 (24.3%)	2,243 (44.5%)	442 (8.8%)	90 (1.8%)	253 (5.0%)

		Home death		Hospital death		Nursing home death		Death in other place [a]
		utilising specialised palliative care services		utilising specialised palliative care services		utilising specialised palliative care services		
Cancer type	Healthcare region	No [a, b]	Yes [a, c]	No [a, d]	Yes [a, e]	No [a, f]	Yes [a, g]	
	Western region	615 (14.4%)	181 (4.2%)	1,779 (41.7%)	440 (10.3%)	855 (20.1%)	347 (8.1%)	46 (1.1%)
	Southeastern region	452 (16.6%)	319 (11.7%)	928 (34.2%)	288 (10.6%)	631 (23.2%)	92 (3.4%)	6 (0.2%)
	Southern region	521 (10.5%)	670 (13.5%)	1,834 (37.1%)	765 (15.5%)	811 (16.4%)	184 (3.7%)	161 (3.3%)
	Total	2,638 (10.7%)	2,478 (10.0%)	9,004 (36.4%)	4,838 (19.5%)	4,305 (17.4%)	896 (3.6%)	608 (2.5%)
Breast & gynaecological	Northern region	168 (10.1%)	177 (10.6%)	549 (32.9%)	156 (9.3%)	438 (26.2%)	100 (6.0%)	83 (5.0%)
	Uppsala-Örebro region	454 (11.7%)	429 (11.1%)	1,147 (29.6%)	732 (18.9%)	1,048 (27.0%)	44 (1.1%)	25 (0.6%)
	Stockholm region	80 (2.2%)	530 (14.4%)	648 (17.7%)	1,703 (46.4%)	467 (12.7%)	70 (1.9%)	173 (4.7%)
	Western region	469 (14.1%)	210 (6.3%)	829 (25.0%)	466 (14.0%)	892 (26.9%)	406 (12.2%)	48 (1.4%)
	Southeastern region	331 (15.9%)	305 (14.6%)	500 (24.0%)	231 (11.1%)	635 (30.4%)	83 (4.0%)	2 (0.1%)
	Southern region	345 (10.5%)	505 (15.3%)	798 (24.2%)	573 (17.4%)	770 (23.4%)	169 (5.1%)	135 (4.1%)
	Total	1,847 (10.3%)	2,156 (12.0%)	4,473 (25.0%)	3,862 (21.5%)	4,250 (23.7%)	872 (4.9%)	466 (2.6%)
Prostate and urinary tract	Northern region	325 (12.2%)	251 (9.4%)	825 (30.9%)	184 (6.9%)	852 (32.0%)	127 (4.8%)	102 (3.8%)
	Uppsala-Örebro region	908 (15.1%)	663 (11.0%)	1,626 (27.1%)	742 (12.4%)	1,986 (33.1%)	56 (0.9%)	21 (0.3%)
	Stockholm region	95 (2.2%)	576 (13.6%)	918 (21.6%)	1,584 (37.3%)	833 (19.6%)	37 (0.9%)	200 (4.7%)
	Western region	794 (17.4%)	211 (4.6%)	1,209 (26.6%)	477 (10.5%)	1,525 (33.5%)	286 (6.3%)	51 (1.1%)
	Southeastern region	566 (19.3%)	332 (11.3%)	665 (22.6%)	223 (7.6%)	1,072 (36.5%)	74 (2.5%)	6 (0.2%)
	Southern region	715 (15.8%)	616 (13.6%)	1,063 (23.5%)	564 (12.5%)	1,342 (29.6%)	134 (3.0%)	93 (2.1%)
	Total	3,405 (13.7%)	2,649 (10.6%)	6,308 (25.3%)	3,774 (15.1%)	7,611 (30.5%)	714 (2.9%)	473 (1.9%)
Haematological	Northern region	87 (6.9%)	74 (5.9%)	679 (54.0%)	79 (6.3%)	258 (20.5%)	61 (4.8%)	20 (1.6%)

Cancer type	Healthcare region	Home death utilising specialised palliative care services: No [a, b]	Home death utilising specialised palliative care services: Yes [a, c]	Hospital death utilising specialised palliative care services: No [a, d]	Hospital death utilising specialised palliative care services: Yes [a, e]	Nursing home death utilising specialised palliative care services: No [a, f]	Nursing home death utilising specialised palliative care services: Yes [a, g]	Death in other place [a]
	Uppsala-Örebro region	277 (9.4%)	214 (7.3%)	1,528 (52.1%)	303 (10.3%)	576 (19.6%)	26 (0.9%)	8 (0.3%)
	Stockholm region	48 (1.9%)	317 (12.7%)	952 (38.1%)	751 (30.1%)	314 (12.6%)	37 (1.5%)	78 (3.1%)
	Western region	284 (11.6%)	109 (4.5%)	1,347 (55.2%)	102 (4.2%)	434 (17.8%)	146 (6.0%)	20 (0.8%)
	Southeastern region	201 (12.6%)	152 (9.5%)	721 (45.3%)	110 (6.9%)	364 (22.8%)	44 (2.8%)	1 (0.1%)
	Southern region	183 (7.3%)	194 (7.7%)	1,321 (52.6%)	252 (10.0%)	448 (17.8%)	66 (2.6%)	49 (1.9%)
	Total	1,080 (8.2%)	1,060 (8.0%)	6,550 (49.5%)	1,598 (12.1%)	2,394 (18.1%)	380 (2.9%)	176 (1.3%)
Malignant melanoma and sarcoma	Northern region	34 (9.0%)	40 (10.6%)	124 (32.9%)	53 (14.1%)	86 (22.8%)	21 (5.6%)	19 (5.0%)
	Uppsala-Örebro region	117 (12.4%)	127 (13.5%)	265 (28.2%)	200 (21.3%)	222 (23.6%)	10 (1.1%)	0 (0.0%)
	Stockholm region	13 (1.7%)	120 (15.6%)	153 (19.8%)	371 (48.1%)	57 (7.4%)	15 (1.9%)	42 (5.4%)
	Western region	144 (17.5%)	44 (5.3%)	231 (28.1%)	121 (14.7%)	173 (21.0%)	98 (11.9%)	12 (1.5%)
	Southeastern region	84 (16.7%)	73 (14.5%)	120 (23.8%)	80 (15.9%)	120 (23.8%)	26 (5.2%)	1 (0.2%)
	Southern region	71 (8.7%)	131 (16.1%)	213 (26.2%)	159 (19.5%)	160 (19.7%)	47 (5.8%)	33 (4.1%)
	Total	463 (10.9%)	535 (12.6%)	1,106 (26.1%)	984 (23.3%)	818 (19.3%)	217 (5.1%)	107 (2.5%)
Other	Northern region	169 (8.6%)	166 (8.5%)	732 (37.4%)	165 (8.4%)	467 (23.9%)	131 (6.7%)	128 (6.5%)
	Uppsala-Örebro region	501 (11.9%)	394 (9.4%)	1,450 (34.5%)	690 (16.4%)	1,101 (26.2%)	48 (1.1%)	23 (0.5%)
	Stockholm region	82 (2.4%)	454 (13.1%)	854 (24.7%)	1,401 (40.4%)	457 (13.2%)	71 (2.0%)	145 (4.2%)
	Western region	560 (16.0%)	190 (5.4%)	1,169 (33.3%)	314 (9.0%)	857 (24.4%)	381 (10.9%)	37 (1.1%)
	Southeastern region	342 (15.6%)	301 (13.8%)	558 (25.5%)	213 (9.7%)	708 (32.4%)	62 (2.8%)	2 (0.1%)
	Southern region	425 (11.7%)	454 (12.5%)	1,160 (31.9%)	512 (14.1%)	852 (23.4%)	125 (3.4%)	111 (3.1%)

Cancer type	Healthcare region	Home death: utilising specialised palliative care services: No [a, b]	Home death: Yes [a, c]	Hospital death: utilising specialised palliative care services: No [a, d]	Hospital death: Yes [a, e]	Nursing home death: utilising specialised palliative care services: No [a, f]	Nursing home death: Yes [a, g]	Death in other place [a]
Total (all cancer types)	Total	2,081 (11.0%)	1,959 (10.3%)	5,927 (31.2%)	3,295 (17.4%)	4,442 (23.4%)	818 (4.3%)	447 (2.4%)
	Northern region	1,620 (10.7%)	1,561 (10.3%)	5,583 (36.9%)	1,314 (8.7%)	3,406 (22.5%)	899 (5.9%)	759 (5.0%)
	Uppsala-Örebro region	4,540 (13.0%)	3,976 (11.4%)	11,588 (33.3%)	5,610 (16.1%)	8,544 (24.6%)	393 (1.1%)	142 (0.4%)
	Stockholm region	666 (2.3%)	4,280 (15.0%)	6,488 (22.8%)	11,934 (41.9%)	3,354 (11.8%)	424 (1.5%)	1,341 (4.7%)
	Western region	4,410 (16.0%)	1,599 (5.8%)	8,973 (32.5%)	3,055 (11.1%)	6,586 (23.9%)	2,663 (9.6%)	325 (1.2%)
	Southeastern region	3,038 (17.4%)	2,448 (14.0%)	4,799 (27.5%)	1,711 (9.8%)	4,836 (27.7%)	624 (3.6%)	25 (0.1%)
	Southern region	3,418 (11.8%)	4,221 (14.6%)	8,636 (29.9%)	4,481 (15.5%)	6,148 (21.3%)	1,105 (3.8%)	891 (3.1%)
	Total	17,698 (11.6%)	18,086 (11.9%)	46,093 (30.2%)	28,113 (18.4%)	32,876 (21.6%)	6,110 (4.0%)	3,486 (2.3%)

Notes. [a] Row percentages and (n). [b] General home care. [c] Specialised palliative home care. [d] Hospital wards in various medical specialities. [e] Specialised palliative care wards. [f] Nursing homes. [g] Municipality hospices

For each place of death per cancer type the healthcare region with the lowest proportion of deaths is highlighted in yellow and the highest proportion is highlighted in blue
