# Supplementary material for: Influence of palliative care policy on place of death for people with different cancer types: a nationwide’ register study
Source: PLoS One. 2025 Mar 27;20(3):e0320086. doi: 10.1371/journal.pone.0320086 (PMC11949374; doi:10.1371/journal.pone.0320086)
Supplement: S3 Table — (PDF) [file pone.0320086.s003.pdf]

Supplementary Table 3. Proportion of i) Home, ii) Hospital, and iii) Nursing home deaths per year and as related to the six healthcare regions

i) Proportion of home deaths

Healthcare region	2013	2014	2015	2016	2017	2018	2019	Change (%) 2013-2019	Percentage points change per year (95%CI)
**Uppsala-Örebro region**	1,083 (22.1%)	1,139 (23.5%)	1,141 (22.9%)	1,272 (25.3%)	1,269 (24.9%)	1,284 (26.1%)	1,328 (26.4%)	+4.3%	0.72 (0.49,0.94)
**Northern region**	415 (20.0%)	427 (19.7%)	456 (20.9%)	483 (22.7%)	441 (20.1%)	491 (22.2%)	468 (21.4%)	+1.4%	0.31 (-0.02,0.63)
**Stockholm region**	657 (16.9%)	652 (16.9%)	736 (17.8%)	782 (19.1%)	722 (17.5%)	700 (16.4%)	697 (16.9%)	0%	-0.06 (-0.28,0.16)
**Western region**	763 (20.3%)	829 (21.3%)	812 (20.8%)	884 (22.7%)	973 (23.4%)	862 (21.6%)	886 (22.2%)	+1,9%	0.32 (0.08,0.56)
**Southeastern region**	737 (29.6%)	742 (29.5%)	774 (31.4%)	764 (31.2%)	830 (32.1%)	774 (31.1%)	865 (34.9%)	+5.3%	0.71 (0.37,1.06)
**Southern region**	975 (23.9%)	1,063 (25.6%)	1,091 (27.2%)	1,090 (25.6%)	1,122 (26.7%)	1,121 (27.6%)	1,177 (28.5%)	+4,6%	0.72 (0.49,0.94)
**Total population**	4,631 (21.8%)	4,853 (22.6%)	5,010 (23.1%)	5,276 (24.1%)	5,360 (24.0%)	5,232 (23.9%)	5,422 (24.7%)	+2.9%	0.42 (0.32, 0.53)

ii) Proportion of hospital deaths

Healthcare region	2013	2014	2015	2016	2017	2018	2019	Change (%) 2013-2019	Percentage points change per year (95%CI)
**Uppsala-Örebro region**	2,381 (48.6%)	2,525 (52.0%)	2,587 (52.0%)	2,497 (49.7%)	2,478 (48.6%)	2,373 (48.3%)	2,357 (46.8%)	-1,8%	-0.59 (-0.85,-0.32)
**Northern region**	955 (46.0%)	1,024 (47.3%)	1,021 (46.7%)	952 (44.7%)	1,036 (47.2%)	948 (42.9%)	961 (44.0%)	-2,0%	-0.51 (-0.91,-0.12)
**Stockholm region**	2,494 (64.3%)	2,489 (64.6%)	2,627 (63.5%)	2,576 (62.9%)	2,742 (66.3%)	2,828 (66.4%)	2,666 (64.6%)	-0,3%	0.28 (-0.00,0.56)
**Western region**	1,656 (44.0%)	1,781 (45.7%)	1,743 (44.6%)	1,768 (45.4%)	1,782 (42.8%)	1,682 (42.1%)	1,616 (40.5%)	-3,5%	-0.70 (-1.00,-0.41)
**Southeastern region**	950 (38.1%)	990 (39.3%)	933 (37.8%)	972 (39.7%)	934 (36.2%)	881 (35.4%)	850 (34.3%)	-3,8%	-0.75 (-1.11,-0.39)
**Southern region**	1,980 (48.4%)	1,735 (41.8%)	1,528 (38.1%)	2,080 (48.9%)	2,014 (47.9%)	1,878 (46.2%)	1,902 (46.0%)	-2,4%	0.40 (0.12,0.69)
**Total population**	10,426 (49.2%)	10,549 (49.2%)	10,443 (48.1%)	10,854 (49.7%)	10,992 (49.1%)	10,590 (48.3%)	10,352 (47.1%)	-2.1%	-0.24 (-0.37,-0.12)

iii) Proportion of nursing home deaths

Healthcare region	2013	2014	2015	2016	2017	2018	2019	Change (%) 2013-2019	Percentage points change per year (95%CI)
**Uppsala-Örebro region**	1,425 (29.1%)	1,184 (24.4%)	1,233 (24.8%)	1,227 (24.4%)	1,324 (26.0%)	1,228 (25.0%)	1,316 (26.1%)	-3,0%	-0.23 (-0.46,0.00)
**Northern region**	597 (28.8%)	622 (28.7%)	596 (27.3%)	567 (26.6%)	598 (27.3%)	661 (29.9%)	664 (30.4%)	+1,6%	0.27 (-0.09,0.63)
**Stockholm region**	580 (14.9%)	559 (14.5%)	586 (14.2%)	561 (13.7%)	506 (12.2%)	480 (11.3%)	506 (12.3%)	-2,6%	-0.59 (-0.79,-0.39)
**Western region**	1,342 (35.7%)	1,282 (32.9%)	1,312 (33.6%)	1,191 (30.6%)	1,359 (32.6%)	1,391 (34.9%)	1,372 (34.4%)	-1,3%	-0.02 (-0.30,0.26)
**Southeastern region**	805 (32.3%)	786 (31.2%)	757 (30.7%)	705 (28.8%)	812 (31.4%)	831 (33.4%)	764 (30.8%)	-1,5%	0.02 (-0.32,0.37)
**Southern region**	1,006 (24.6%)	1,270 (30.6%)	1,344 (33.5%)	939 (22.1%)	897 (21.3%)	931 (22.9%)	866 (20.9%)	-3,7%	-1.38 (-1.63,-1.13)
**Total population**	5,757 (27.1%)	5,704 (26.6%)	5,828 (26.9%)	5,190 (23.7%)	5,497 (24.6%)	5,522 (25.2%)	5,488 (25.0%)	-2.1%	-0.41 (-0.52,-0.30)

Note. CI, confidence interval.
