# Supplementary material for: Influence of palliative care policy on place of death for people with different cancer types: a nationwide’ register study
Source: PLoS One. 2025 Mar 27;20(3):e0320086. doi: 10.1371/journal.pone.0320086 (PMC11949374; doi:10.1371/journal.pone.0320086)
Supplement: S4 Table — (PDF) [file pone.0320086.s004.pdf]

Supplementary Table 4. Interaction between longitudinal trends in place of death and covariates for a) people residing in own homes aged 18 and older, and for b) people residing in nursing homes aged 60 and older

	People residing in own homes			People residing in nursing homes		
**Covariates**	**Percentage points change per year (95%CI)[a]**	**Covariate*Time interaction**	**R^2 at aggregated level [b]**	**Percentage points change per year (95%CI)[a]**	**Covariate*Time interaction**	**R^2 at aggregated level [b]**
**Sex**		p=0.73	0.95		p=0.57	0.69
Male	-0.49 (-0.69,-0.29)			0.28 (-0.48,1.04)		
Female	-0.54 (-0.75,-0.33)			0.56 (-0.06,1.19)		
**Age at death years**		p=0.004	0.48		p=0.82	0.85
18-29	-0.40 (-2.87,2.06)			N/A		
30-39	1.40 (-0.20,3.00)			N/A		
40-49	0.23 (-0.66,1.12)			N/A		
50-59	0.04 (-0.47,0.55)			N/A		
60-69	-0.52 (-0.83,-0.21)			1.44 (-0.94,3.82)		
70-79	-0.40 (-0.64,-0.16)			0.53 (-0.85,1.90)		
80-89	-0.71 (-1.00,-0.43)			0.35 (-0.39,1.08)		
90+	-1.26 (-1.88,-0.64)			0.27 (-0.46,0.99)		
**Cancer type**		p=0.035	0.97		p=0.038	0.89
Lower gastrointestinal	-0.29 (-0.72,0.15)			-0.33 (-1.62,0.96)		
Upper gastrointestinal	-0.50 (-0.82,-0.17)			1.91 (0.45,3.38)		
Pulmonary	-0.65 (-0.98,-0.32)			0.55 (-1.23,2.34)		
Breast & gynaecological	-0.04 (-0.47,0.39)			1.00 (0.05,1.94)		
Prostate and urinary tract	-0.87 (-1.26,-0.48)			0.30 (-0.63,1.23)		

Covariates	People residing in own homes: Percentage points change per year (95%CI)[a]	Covariate*Time interaction	R^2 at aggregated level [b]	People residing in nursing homes: Percentage points change per year (95%CI)[a]	Covariate*Time interaction	R^2 at aggregated level [b]
Haematological	-0.60 (-1.00,-0.19)			1.23 (-0.52,2.98)		
Malignant melanoma and sarcoma	0.49 (-0.36,1.34)			-1.84 (-4.42,0.74)		
Other	-0.54 (-0.96,-0.13)			-1.06 (-2.46,0.34)		
**Palliative care diagnosis** [c]		p<.0001	0.91		p=0.005	0.90
No	-1.08 (-1.25,-0.90)			-0.04 (-0.52,0.44)		
Yes	0.68 (0.42,0.93)			2.65 (0.82,4.48)		
**Marital status**		p<.0001	0.98		p=0.31	0.84
Married	-0.29 (-0.49,-0.08)			0.97 (-0.49,2.44)		
Unmarried	-0.48 (-0.87,-0.09)			0.53 (-0.75,1.81)		
Widowed	-1.19 (-1.51,-0.87)			0.52 (-0.11,1.14)		
Divorced	-0.68 (-1.01,-0.35)			-0.60 (-1.78,0.57)		
**Healthcare Region**		p<.0001	0.96		p=0.065	0.89
Uppsala-Örebro region	-0.89 (-1.19,-0.59)			-0.55 (-1.56,0.46)		
Northern region	-0.53 (-1.00,-0.06)			0.50 (-0.79,1.80)		
Stockholm region	0.12 (-0.15,0.40)			1.48 (0.12,2.83)		
Western region	-0.74 (-1.10,-0.38)			1.29 (0.16,2.42)		
Southeastern region	-1.02 (-1.48,-0.56)			-0.33 (-1.47,0.81)		
Southern region	-0.35 (-0.69,-0.02)			-0.11 (-1.16,0.93)		
**Specialised palliative service**[d]		p<.0001	0.99		p<.0001	1.00
No	-1.55 (-1.73,-1.37)			-0.06 (-0.52,0.40)		

	People residing in own homes			People residing in nursing homes		
Covariates	Percentage points change per year (95%CI)[a]	Covariate*Time interaction	R^2 at aggregated level [b]	Percentage points change per year (95%CI)[a]	Covariate*Time interaction	R^2 at aggregated level [b]
Yes	1.18 (0.95,1.41)			5.07 (3.35,6.79)		

Notes. [a] CI: confidence interval. [b] Calculated using linear regression on observed relative frequencies vs time.
