# Supplementary material for: Influence of palliative care policy on place of death for people with different cancer types: a nationwide’ register study
Source: PLoS One. 2025 Mar 27;20(3):e0320086. doi: 10.1371/journal.pone.0320086 (PMC11949374; doi:10.1371/journal.pone.0320086)
Supplement: Attachment — Submitted filename: Response to reviewers.doc [file pone.0320086.s006.doc]

Authors’ response to the reviewers’ comments and amendments made

Manuscript reference number PONE-D-24-38475; with the updated title “Influence of palliative care policy on place of death for people with different cancer types: a nationwide register study”

A special thanks to the reviewer and editor for constructive and encouraging critique of our manuscript. Below you find our considerations as related to the specific points raised.

| **Comments from reviewers** | **Authors’ responses** | **Amendments made in the manuscript** |
| --- | --- | --- |
| **Reviewer #1.** I thank the Academic Editor for giving me the opportunity to read the manuscript “Impact of a national palliative care policy on place of death for people with different cancer types: a national population-based study in a high-income country with an increasing proportion of older people” by Joakim Öhlén and Colleagues (PONE-D-24-38475). There follow some suggestions that I suppose may be helpful to improve the manuscript. I have numbered the pages starting with 1 on the title page.  **Editor.** This is an interesting paper assessing the impact of palliative care policy on the place of death in a large country. Results on the same data analyzing trends in the same period were already published (ref 26). In this manuscript, the authors referred to cancer types. This might be an interesting sub-analysis of the data. The presentation of the data follows the previous pattern, as in the tables and statistical methodology. An effort to compare the total and cancer-specific cancers might probably confirm with this analysis (and some information in the paper about cardiovascular conditions) that major determinants are contextual (area, age groups, SES, etc) more than attributable to specific pathology (with exception also from literature for hematological neoplasms). | Thank you. | No amendments here. |
| **Reviewer #1.** I suggest the authors to consider the possibility to modify it. The term ‘impact’ seems inappropriate, because the authors do not have historical reference data. Also, the country of origin of the paper needs to be mentioned. Conversely, I think that the notation “ ... in a high-income country with an increasing proportion of older people” is rather unnecessary. Besides, patient age is not the only factor being investigated in the study. | We have considered this comment in relation to the hypothesis for the study, and understand ‘influence’ would be a more appropriate term instead of ‘impact’.  We are not totally convinced about stating the name of the contry of origin for the study in the title. We have, especially in worksphs led by editors-in-chief for competetive and influencial international research journals, learned about the advice to avoid including specific country names in a title, but including it in the abstract. The motives have been to increase interest for papers to be of broader interests than for individual countries.  We agree to omit older people in the title. | We have revised the title to:  Influence of palliative care policy on place of death for people with different cancer types: a nationwide’register study |
| **Reviewer #1.** Page 6. “ … living in rural areas is associated with hospital deaths [13]”.  Please note that, in the next sentence, an opposite association is mentioned (rural areas and home deaths). I suppose that there are inconsistencies between the results of cited studies. | Thanks for noticing this. | We have primarily built on a literature review (reference #16), and added that there are contrary results reported. |
| **Reviewer #1.** Page 7. Last sentence of the top paragraph. This is a strong and important statement. Does it have some relationship with the next one (see below)?  Page 7. Bottom paragraph.  The statement “ … the hypothesis is that end-of-life and palliative care policy shape healthcare services, which in turn influence service utilization and ultimately place of death” seems to be central to the rationale of the study but is too concise and somewhat unclear. In particular, it is unclear to me what “ … end-of-life and palliative care policy shape healthcare services …” means. The study hypothesis needs to be explained better. The same statement is in the Abstract too. Perhaps a brief clarification of terms can be added. | We have considered a clarification to be informed by the theoreticl source (reference #24) we have used for the hypothesis. Accordingly, we can clarify ‘healthcare services’ as ‘types, locations and practices’. | We have added that “… palliative care policy shape healthcare service types, locations and practices …” |
| **Reviewer #1.** Page 8. Lines 3-6.  “… recent place of death result for the total population”. Do the authors mean “… results of a recent study on the place of death for the total population”? | Yes, this needs to be reformulated. | We have changed to : “…results of a recent study on place of death for the total population …”. |
| **Reviewer #1.** Page 8. Bottom line.  Please include ‘nationwide’ in the study definition. Also, although two references are provided to the reader, a very concise description of the national guidelines of 2012 would be useful. | Thanks. | ‘Nation-wide’ is added in the study definition.  Brief explanations of the two national palliative care policy documents are added. |
| **Reviewer #1.** Page 9. Top paragraph.  Record linkage procedures are an important methodological issue. Have the authors used a personal universal or unique identifying number? If so, I do not understand the need for anonymising the records. Some more detail of the health record linkage procedures that are available in Sweden would be of interest to the reader. | The peersonal identity numbers of the deceased individuals were replaced by serie numbers by the National Board of Health and Welfare, i.e. the register holder of the death certificate data and the authority who linked data from all the registers involved. According to the Swedish legistlation as related to the current data set, strict confidentiality applies to this type of data pertaining personal information, even for the deceased. The unique data set was detroyed by the National Board of Health and Welfare three months after we received thea data set. Therefore, any new data access needs to forego ethical clearence and a specific application to the register holders respectively.  Since we are fully aware of the “Open Data” principle, we have consultet Swedish National Data Service and the National Board of Health and Welfare and our updated statement is based on their advice. | We have revised the description of data linked in the section “Desigh” under Materials and methods. Addresses to the register holders are added in the section “Data Availability”. |
| **Reviewer #1.** Page 10. Line 3.  Perhaps Beverage should be changed to Beveridge. | Thanks for noticing the misspelling. | Changed to ‘Beveridge’. |
| **Reviewer #1.** Page 11. Study variables.  The place of death was classified into “hospital, home, nursing home, other”. In the footnote to Table 1 (page 15), it reads that specialised palliative services included specialised palliative home care, specialised palliative hospital care, and municipality hospice care. This seems to suggest that there could also be non-specialised palliative home care services and non-specialised palliative hospital care services. Can the authors provide some details or examples? Secondly, in which category of the four-tier classification are municipal hospices classified? The nomenclature used should be clarified and completed in the body of the text (the reader would notice the footnote to Table 1 too late …). Finally, please clarify “… except from other places …”. | Thanks for the suggestions. | We have added the nomenclature in the footnote of table 1, and added a clarification at the end of the “Study variables” section. We have clarified that distiguishing specialised palliative sercvices or not did not apply to ‘Other places of death’ |
| **Reviewer #1.** Page 17 (and elsewhere). “… palliative care diagnosis …”. Unfortunately, I do not know the meaning of this term. Please provide a definition.  **Editor**. palliative care diagnosis (it needs a better explanation, as requested by the reviewer) | The diagnose code for ‘palliative care’ is a specific diagnsotic code included in the International Statistical Classification of Diseases and Related Health Problems (ICD). However, to our knowledge, there are only brief explanations of this term available and also guidance lacking, which our results also reflect. We had already in the previous version of the manuscript included a comment on this in the discussion. | Clarifications to the diagnsosis ‘palliative care’ is added a) briefly in the Study variables section and b) in a footnote to table 1. |
| **Reviewer #1.** In Table 3, the columns headed ‘No’ are very difficult to interpret. The sum of the row percentages is 100%. Apparently, this means that –for example– a person who did not die at home (first column) could not die elsewhere. Of course, this cannot be the case. Please clarify. | Thanks for pointing this out; we agree.  We now recognised the labels of the columns create a misunderstanding; the row percentages should be 100%; any individual can only be included in one of the cells. | Iwe have made chages in table 3:   - Wordings in the labels of the columns - Clarifications to ‘No’ for death in ‘specialised palliative services’ (i.e. Other home / hospital / nursing home death) are added in footnotes. |
| **Reviewer #1.** Page 28. Bottom lines.  The age groups 80-89 years and 90+ showed a downward trend to die in hospital. Was this decrease associated with an increasing trend to die at home? This is probably shown in the Figure, but the resolution in the PDF is not good. If an increasing trend to die at home was observed in the elderly, this (unexpected, I guess) finding would need a comment. | Figure 3 shows the percentage change for those residing in own homes to die in hospitals as compared at home, and yes, there was an increasing trend for the patients aged 60 and older to die at home; however, the trends for these odlest age groups were small and varied from 0.38 to 0.95 percentage change. Notably, one out of five for those 80 years and older died at home. | The percentage changes for the two oldest age groups are added (p. 29).  We have added considerations of the proportion of nursing home deaths in a new paragraph discussing the influence of age in the Discussion. |
| **Reviewer #1.** Page 31. Lines 1-4.  Please explain more explicitly why the study hypothesis was not confirmed. | We agree. | Clarifications added in the first para of the Discussion. |
| **Reviewer #1.** Page 32. Line 6.  “… decreased from 20.6% in 1023 to 23.4% in 2019…”. In fact, this is an increase. | Thanks for noticing this. | Changed to ‘increased’. |
| **Editor:** Sweden has a high proportion of deaths in nursing homes and the proportion is stable and high compared with other countries. So, this may explain the slow change in-hospital deaths after 2012.  **Editor.** The study investigated how the introduction of palliative care diagnosis (it needs a better explanation, as requested by the reviewer) in different settings (in-hospital palliative care) might reduce the need to transfer the cancer patient to home. At leat in my knowledge palliative care was mainly at home. | The high proportion of nursing home deaths in Sweden is disctinct. However, it is worth noticing that the proportion of people in the cancer population dying in nursing homes was lower (25.6%) as compared to the general population during the same years (37.7%; reference #26).  We are doubtful for the palliative care diagnosis to reduce the need for transferring patients to home care. Rather, in the Swedish case, our result can be an expression for hospital focused care. | We have added considerations of the proportion of nursing home deaths in a new para discussing the influence of age in the Discussion.  No additional changes made. |
| **Reviewer #1.** Page 33. Middle paragraph.  “… comparatively great regional and municipal freedom in organising services …”. I suggest the Authors to discuss more deeply this key issue. | Thanks. | Further elaboration added. |
| **Editor.** Finally, possibly a more in-depth knowledge of the last year of life is important. In recent years, the treatment of metastatic cancer has changed and possibly is much more medicalized than before. Perhaps, a better knowledge of the end-of-life pattern of oncological care should be relevant in explaining the place of death. | We agree. | The Discussion related to early integrated palliative care is expanded about consequences of new and changed oncological treatments possible for advanced cancer and the diverse patient illness trajectories at the end of life. |
| **Reviewer #1.** Page 34..  I agree that the exclusion of the years prior to the introduction of the new Guidelines is a limitation of the study. However, this does not mean that a sound conclusion cannot be drawn from the data. The suboptimal situation observed, indeed, points to the need for an improvement. From this point of view, the problem of the relatively limited time span of the study is mitigated by a consideration: this study was undertaken in a timely manner. In addition, the results are disappointing enough to represent a baseline from which to improve.  **Editor**. The trend considered in this paper is 2013-2019, after the 2012 recommendations and innovations in policy. There is no reference to the possible existence of a trend before 2012. For example, in a study in Germany from 2001 to 2011, there was a decreasing trend in this period; certainly, the issue of hospital deaths was at the center of interest before 2012, and a decreasing trend might have started after 2000. | We have considered these two thoughtful comments together. In fact, in Sweden there were no national policy in place related to palliative care before 2013. There were governmental initiated investigations, but no decisions made based on these reports. As a result, the palliative care development before 2013 were primarily bottom up driven by health professionals. Although the development involved palliative care in home care services, there were large efforts implemented in hospitals. However, there are no data describing the historical development, and unclear to what extent it influenced place of death in the cancer population. | We have in the section on “Strengths and limitations” in the Discussion clarified that we have tried to mitigate through methodological and theoretical considerations. |
| **Reviewer #1.** Page 36. “… the need for improved national strategies for palliative cancer care …” and “... a great need for national strategies for governance …”.  An improvement of palliative care for cancer is undoubtedly needed, but I suggest the authors to centre their conclusions on the issue of the effectiveness of Guidelines of 2012, in line with the rationale of the study. For example, the results might be interpreted to show (1) that a revision of the national policy is needed or (2) that there is only a problem of insufficient implementation or (3) that it is premature to make a decision about the policy and that other years of monitoring are necessary – and so on. Also, assuming that the authors support a revision, which priorities does the study suggest (if any)? | We agree. | We have revised the conclusion to making it focused on the influence of the national policy, i.e. strengthening the conclusion in relation to the study aim and hypothesis. |
